# How Much Can We Bet on Activity of BET Inhibitors Beyond NUT–Midline Carcinoma?

**DOI:** 10.1093/jncics/pkz092

**Published:** 2019-11-06

**Authors:** Patricia Martin-Romano, Capucine Baldini, Sophie Postel-Vinay

**Affiliations:** 1 DITEP (Département d’Innovations Thérapeutiques et Essais Précoces), Gustave Roussy, Villejuif, France; 2 Université Paris Saclay, Université Paris-Sud, Faculté de médicine, Le Kremlin Bicêtre, France; 3 ATIP-Avenir group, Inserm Unit U981, Gustave Roussy, Villejuif, France

Epigenetic regulation involves more than 800 epigenetic enzymes that act at multiple levels in an orchestrated fashion to regulate gene expression. These can be grouped schematically into four categories: writers, readers, erasers, and shapers. The Bromodomain Extra-Terminal (BET) family of readers, which includes BRD2, BRD3, BRD4, and BRDT, recognize acetylated lysine on histones—a mark associated with active transcription at promotors, enhancers, and super-enhancers—and recruit transcription factors (1). BET proteins play an important role in cancer, notably by promoting aberrant expression of *MYC* or other oncogenes, or throughout oncogenic rearrangements, such as the *BRD4-NUMT1* translocation, which drive NUT–midline carcinoma (NMC), an extremely aggressive undifferentiated squamous cell carcinoma (2–4).

Over the last few years, the development of clinical-grade small molecule BET inhibitors (BETi) has generated great enthusiasm, with the perspective of “drugging the undruggable,” Following very encouraging initial preclinical activity in *MYC*–driven diseases and clinical activity in NMC (5,6), multiple BETi have been developed, which differ by their chemical characteristics (notably affinity toward certain bromodomains and mono versus bivalent BETi [1,7]).

Here, Piha-Paul et al. report the results of the phase I dose-escalation study of molibresib (GSK525762) in 65 patients with NMC and other solid tumors (8). Once-daily molibresib was tolerated at doses demonstrating target engagement, and 80 mg once daily was selected as the recommended phase II dose. Consistent with known class effects of BETi, most frequent adverse events were hematological toxicities (mostly thrombocytopenia), gastrointestinal toxicities, and fatigue. Nineteen patients with NMC were enrolled, representing the largest NMC prospective series enrolled in a BETi phase I trial so far (Table 1). Among those, four patients (22%) experienced a partial response (PR), two of which were confirmed. No confirmed response was observed among the 46 patients with other tumor types.

**Table 1.** Clinical trials evaluating BET inhibitors with results in patients with solid tumors*

Clinical trial	Design	Drug	Patients, No.	Overall response rate	Median time of study	Toxicity	Recommended dose	Publication
NCT01949883	3 + 3, escalation	CPI-0610	64 lymphoma pts DLBCL *n* = 36 (56%) FL *n* = 8 (12.5%) HL *n* = 5 (8%) MCL *n =* 3 (5%) Other *n* = 12 (19%)	38 evaluable pts CR 2 pts PR 3 pts SD 17 pts	NA	G3 rash *n* = 1 Neutropenia *n* = 1 Diarrhea *n*=2 G4 thrombocytopenia *n* = 3	Safety and PK data, 225 mg	TAT 2018, (23)
NCT02259114	3 + 3, escalation	MK-862 (birabresib)	46 pts NUT *n* = 10 (22%) CRPC *n* = 26 (57%) NSCLC *n* = 10 (22%)	42 evaluable pts CR 0 PR 3 NMC pts (7%) SD *n* = 25 (60%) NMC *n* = 3 CRPC *n* = 15 NSCLC *n* = 7	2.3 m (0.2–15.4 m) PR (NMC), 1.4–8.4 m, 1 after 10 m SD CRPC *n* = 2: 3.5 m, 7.8 m NSCLC *n*	G3–4 nausea *n* = 1 vomiting *n* = 1 fatigue *n* = 2 anemia *n* = 11 thrombocytopenia *n* = 20 ALT *n* = 2 Acute kidney injury *n* = 1 Thrombocytopenia nadir 32 d (range = 12–211)	PK data, 80 mg	Lewin 2018 (9)
NCT02683395	3 + 3, escalation	PLX51107	36 pts Uveal melanoma *n* = 11 Sarcoma *n* = 6 NSCLC *n* = 2 Breast *n* = 2 CRPC *n* = 2	36 evaluable pts SD *n* = 8 Uveal Melanoma *n* = 2 Sarcoma *n* = 3 NSCLC *n* = 1 CRPC *n* = 1	4–14 mo	G3 Nausea *n* = 1 Thrombocytopenia *n* = 1	Preclinical toxicology data	Patnaik et al, JCO 2018 (23)
NCT02391480	3 + 3, escalation	ABBV-075	72 pts Uveal Melanoma *n* = 10 Breast *n* = 8 Pancreatic *n* = 6 HNSCC *n* = 5 CRPC *n* = 3 Others *n* = 40	65 evaluable pts SD *n* = 25 (39%) PD *n* = 40 (61%)	7.6 w (range = 0.9–39.6 wk)	G3–4 Thrombocytopenia *n* = 16 Anemia *n* = 8	Safety	Piha P. et al, 2019 (24)
NCT02711137	3 + 3, escalation	INCB057643	16 pts Solid tumors *N* = 13 Lymphoma (FL) *N* = 3	11 evaluable pts PR *n* = 1 lymphoma (8 m) SD Biliary Cancer *n* = 1 >6 m; Solid tumor *n* = 4 <6 m PD 6 pts	59.5 d (range = 6–282) d	G3–4 Thrombocytopenia *n* = 2 (13%) Anemia *n* = 1 (6%) Bilirubin *n* = 1 (6%) Hyperglycemia *n* = 1 (6%) INR *n* = 1 (6%)	DLTs during cycle 1	Falchook G. et al, 2019 (25)
NCT02516553	Bayesian logistic regression model Arm A: continuous regimen Arm B: 14 d on/7 d off	BI 894999	46 pts Arm A *n* = 21 Arm B *n* = 16	36 evaluable pts PR *n* = 2 (6%) small bowel & esophageal SCC SD 14 (39%) *n* =2 ≥ 4 cycles	2 cycles (range, 1–14) PR 1 pt cycle 2–14 1 pt cycle 2–8	G3–4 Thrombocytopenia *n* = 24 (65%) Fatigue *n* = 3 Diarrhea *n* = 5	Safety DLT, MTD	Bechter O. et al, 2018 (26)
NCT02419417	3 schedules: A (5 d on/2 d off) B (14 d on/7 d off) C (7 d on/14 d off)	BMS 986158	75 pts NUT *n* = 4 Other solid tumor	1 pt NUT (schedule A) 279 d, SD -16%	1 pt with SD, 279 d (9.3 months)	G3–4 Thrombocytopenia *n* = 10 (15%) Fatigue *n* =1 (1%) Nausea 1 pt (1%)	Safety and PK data	Hilton J. et al, 2018 (27)
NCT02369029	Adaptive design: dose escalation MTD - solid tumors expansion at MTD solid dose level dose escalation in hematologic + expansion at MTD hematologic	BAY 17437	8 pts Terminated because of toxicity CRC *n* = 3 NET *n* = 1 Ovarian *n* = 1 CRPC *n* =1 Thyroid *n* = 1 Rectal NET *n* = 1	8 pts SD *n* = 2 pts, 6 cycles	NA	G3-4 Headache *n* = 3 Vomiting *n* = 2 (25%)	Safety and PK data	Postel-Vinay S. et al, 2018 (28)
NCT01587703	3 + 3, escalation	GSK525762	65 pts CRC *n* = 22 NMC *n* = 11 CRPC *n* = 9 SCLC *n* = 6 Breast *n* = 5 NSCLC *n* = 2 Neuroblast *n* = 1 Myeloblast *n* = 1	NC cohort *n* = 19 PR *n* = 2 (11%) SD *n* = 8 Non-NMC *n* = 41 uPR Breast *n* = 1 SD > 4 m CRPC, CRC	NMC median PFS 2.5 m	G3–4 Thrombocytopenia *n* = 24 Nausea *n* = 2 Anorexia *n* = 3 Vomiting *n* = 1 Anemia *n* = 5 Br *n* = 3 Fatigue *n* = 3	Safety and PK data	Piha-Paul S. et al, 2018 (8)

*CRC = colorectal cancer; CRPC = castration-resistant prostate cancer; DLBCL, *n* = 36 (56%); DLT = dose-limiting toxicity; ENA = EORTC-NCI-AACR; FL = follicular lymphoma; HL = Hodgkin lymphoma; HNSCC = Head and Neck Squamous Cell Carcinoma; MCL = mantle cell lymphoma; MTD = maximum tolerated dose; NET = neuroendocrine tumor; NMC = NUT–midline carcinoma; NSCLC = Non-Small Cell Lung Cancer; PK = pharmacokinetics; pts = patients; PR = partial response; SCLC = Small Cell Lung Cancer; SD = stable disease; uPR = unconfirmed partial response.

With more than 15 clinical-grade BETi in early phase development, what can we learn from the results of the molibresib phase I trial? Unsurprisingly, antitumor efficacy was observed almost exclusively in NMC, providing the proof-of-concept for molibresib on-target activity. Interestingly, all four NMC who remained on treatment longer than 6 months had nonthoracic primary tumors, and three of four patients who presented PR had tumors harboring the *BRD3-NUTM1* fusion; the limited size of the series precluded from correlating fusion gene status with treatment duration. BETi antitumor activity has been observed in NMC patients with both *BRD4-NUT* and the less common non–*BRD4-NUT* fusions, such as *BRD3-NUT* and *NSD3-NUT* fusions. For example, responses to birabresib were mostly observed in patients with *BRD4-NUT* fusions (5,9), whereas the patient who presented tumor shrinkage and 9-month clinical benefit on BMS-986158 had a *BRD3-NUT* fusion (10). It is noteworthy that the novel prognostic classification for NMC that is described in an accompanying article of this *JNCI Cancer Spectrum* issue reports that, among nonthoracic primaries, non-*BRD4-NUT* fusions are associated with improved outcome (11). Whether NMC harboring non–*BRD4-NUT* fusions have a distinct biology and are more likely to be sensitive to certain BETi deserves further exploration.

The original work that established the efficacy of BETi in NMC preclinical models showed that JQ1 could displace BRD4-NUT from chromatin, resulting in phenotypic squamous cell differentiation and growth arrest (6). Although a similar process seems to operate in human tumors, as suggested by histopathological changes observed in a posttreatment biopsy of one of the first responding tumors (5), additional mechanisms may occur. Indeed, most responses to BETi in NMC are rapid and short-lived, and followed by a quick onset of resistance; in contrast, some patients presented delayed or prolonged responses, occurring up to 8 months after therapy initiation and lasting 15 months (9)—kinetics that more closely mirror what has been observed in some hematologic malignancies (12)—this suggests that a rapid oncogene “de-addiction” phenomenon, responsible for quick responses, may co-occur alongside the cell differentiation mechanism, rather underlying delayed responses. In this context, investigating and understanding resistance mechanisms to BETi in NMC is essential. Various resistance mechanisms have been described, including BRD4 protein accumulation in Burkitt lymphoma (13), transcriptional plasticity favoring compensatory upregulation of *MYC* through the Wnt/beta-catenin signaling activation in acute myeloid leukemia (14), kinome reprogramming in ovarian cancer (15), and hyperphosphorylation of BRD4 in triple-negative breast cancer (16). This plethora of mechanisms, which may be cell type–specific, and the absence of so-called gatekeeper mutations of the target itself illustrates the complexity of BETi resistance. In this context, further investigation of resistance mechanisms is required to understand the precise mechanism of action of BETi, to develop predictive biomarkers of response—which are still crucially lacking—and to propose rational combinatorial therapies to increase long-term efficacy of BETi.

The relative lack of efficacy of molibresib outside NMC, even in *MYC*–driven diseases such as neuroblastoma, is concerning. This is not an isolated case among BETi (Table 1): Despite preclinical rationale, most BETi have failed to show efficacy in *MYC*–driven diseases, calling for further pharmacodynamic and biomarker investigations. Although target engagement was demonstrated in surrogate tissue with modulation of circulating MCP-1 with molibresib, the level of target modulation achieved in the tumor is unknown. This information, however, is of critical importance, and we can hope that on-treatment tumor biopsies will be collected during phase II trials. Indeed, the lack of efficacy observed outside of NMC may be due to insufficient target modulation in the tumor. How could this challenge be addressed? Based on current safety data, higher molibresib doses are not tolerable, and the 80 mg recommended phase II dose—which led to 50% of grade 3–4 thrombocytopenia, 22% of treatment interruptions, and 16% of dose reductions in a highly selected phase I patient population—might already be challenging in an all-comer patient population. The choice of this high dose was sound, as decreasing the dose in a given patient is always feasible and because intermittent schedules might have negatively affected molibresib efficacy, considering its very short half-life (3–7 h). In this context, targeted drug delivery strategies (such as liposomal formulations of antibody-drug conjugates) might allow an increase of the therapeutic window. Heterobifunctional small molecule BET protein degraders, such as the proteolysis-targeting chimera (PROTAC) ARV-771, ARV-825, BETd-260/ZBC260, dBET6, or QCA570 (13,17–19), also represent attractive future options (20). Whether these will allow increasing the therapeutic window remains to be evaluated in patients, and the results of the first trials evaluating PROTACs will bring essential information in this regard (NCT03888612).

Finally, it is likely that the greatest potential from BETi will arise from combination strategies, using BETi as a “potentiator” (eg, with cytotoxic therapies), or to reverse or delay acquired resistance (eg, in combination with hormonal therapies for breast and prostate cancer, or with kinase inhibitors to counteract kinase reprogramming) (reviewed in 21). These strategies are being investigated in multiple ongoing clinical trials (Figure 1). We hope that, together with appropriate biomarker studies, these trials will help to unlock the full potential of BETi.

**Figure 1.**
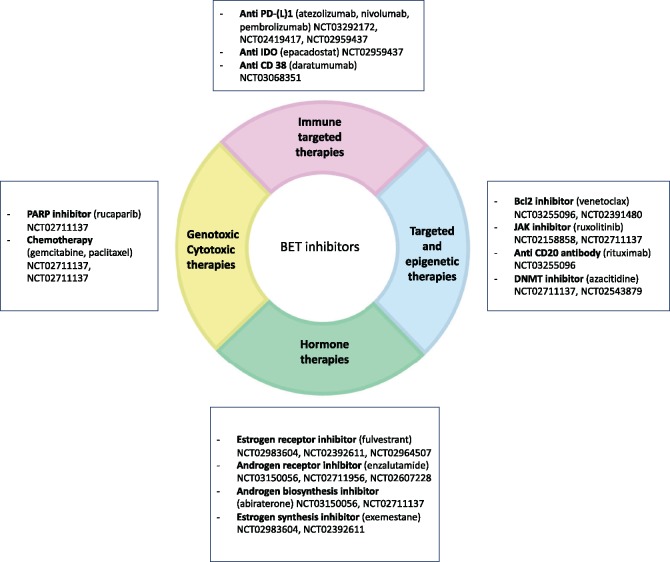
Combination therapies with Bromodomain Extra-Terminal (BET) inhibitors: ongoing clinical trials.

## Notes

Affiliations of authors: DITEP (Département d’Innovations Thérapeutiques et Essais Précoces), Gustave Roussy, Villejuif, France (PM-R, CB, SP-Y); Université Paris Saclay, Université Paris-Sud, Faculté de médicine, Le Kremlin Bicêtre, France (SP-Y); ATIP-Avenir group, Inserm Unit U981, Gustave Roussy, Villejuif, France (SP-Y).

SPV INSERM laboratory is funded by the INSERM ATIP-Avenir grant and Integrated Cancer Research Site (SIRIC) SOCRATE-2 INCa-DGOS-INSERM_12551.

**Conflict of interest statement:** As part of the Drug Development Department (DITEP), PMR, CB, and SPV are principal investigator or sub investigator of clinical trials from Abbvie, Agios Pharmaceuticals, Amgen, Argen-X Bvba, Arno Therapeutics, Astex Pharmaceuticals, Astra Zeneca, Aveo, Bayer Healthcare Ag, Bbb Technologies Bv, Blueprint Medicines, Boehringer Ingelheim, Bristol Myers Squibb, Celgene Corporation, Chugai Pharmaceutical Co., Clovis Oncology, Daiichi Sankyo, Debiopharm S.A., Eisai, Eli Lilly, Exelixis, Forma, Gamamabs, Genentech, Inc., Glaxosmithkline, H3 Biomedicine, Inc., Hoffmann La Roche Ag, Innate Pharma, Iris Servier, Janssen Cilag, Kyowa Kirin Pharm. Dev., Inc., Loxo Oncology, Lytix Biopharma As, Medimmune, Menarini Ricerche, Merck Sharp & Dohme Chibret, Merrimack Pharmaceuticals, Merus, Millennium Pharmaceuticals, Nanobiotix, Nektar Therapeutics, Novartis Pharma, Octimet Oncology Nv, Oncoethix, Onyx Therapeutics, Orion Pharma, Oryzon Genomics, Pfizer, Pharma Mar, Pierre Fabre, Roche, Sanofi Aventis, Taiho Pharma, Tesaro Inc, and Xencor. SPV has participated in advisory boards for Merck KGaA; has benefited from reimbursement for attending symposia from AstraZeneca; and has received laboratory research funding from Fondation Roche France, Boehringher Ingelheim, and Merck KGaA. CB received personal fees from BMS, Sanofi, Abbvie, and Astra Zeneca.
